# Feasibility study on recycled vegetable oil waste and recycled polyethylene for the modification of aged asphalt

**DOI:** 10.1371/journal.pone.0244159

**Published:** 2021-01-05

**Authors:** Xiangqian Ye, Xiaoling Zou, Fafu Tian, Honglin He

**Affiliations:** 1 School of Civil Engineering, Chongqing Jiaotong University, Chongqing, China; 2 National and Local Joint Engineering Laboratory of Traffic Civil Engineering Materials, Chongqing Jiaotong University, Chongqing, China; Mirpur University of Science and Technology, PAKISTAN

## Abstract

The application of reclaimed asphalt pavement has been widely encouraged due to its significant economic and environmental benefits. However, it is necessary to add rejuvenators to ensure its performance. Currently, bio-oil-based regenerants have attracted attention owing to their advantages of renewability and cost savings. The purpose of this paper is to study the use of recycled vegetable oil waste (R-oil) and recycled polyethylene particles for the regeneration and modification of aged asphalt. Physical, rheological, and chemical tests were used to figure out their influence on the pavement performance of aged asphalt. According to the physical test indices (penetration, softening point, and ductility), the performance of the rejuvenated asphalt was better than that of virgin asphalt. The workability and low-temperature performance of the rejuvenated asphalt were basically the same as those of virgin asphalt, and its fatigue and high-temperature performance were better. Infrared spectroscopy showed that R-oil diluted the high-polarity sulfoxide base of aged asphalt. Gel permeation chromatography showed that its molecular weight dispersion was better than that of aged asphalt. Therefore, R-oil and polyethylene can improve the pavement performance and chemical properties of aged asphalt.

## Introduction

Currently, due to the continuous development of highway construction, a large amount of old asphalt mixture are produced in the process of new road construction, remodeling, or the demolition of road structures, which aggravates the impact of solid waste on the global environment [1–5]. In the past few years, reclaimed asphalt pavement (RAP) has attracted comprehensive attention owing to its advantage of supporting a green economy [6–8]. Through the Long-Term Pavement Performance observation project, the National Center for Asphalt Technology found that the pavement performance of recycled asphalt pavement containing 30% RAP is roughly the same as that of ordinary asphalt pavement and that construction costs can be reduced by about 27% [9,10]. The use of RAP materials can also reduce the exploitation of stone and oil. This can save energy and reduce emissions and save the space that would be used for waste landfill [11–13]. Owing to long-term exposure to heat, oxygen, and ultraviolet radiation, the aging of asphalt in RAP material is more serious. In particular, the fatigue performance is significantly reduced [14–16]. Therefore, it is necessary to add a certain proportion of regenerant and modifier to restore its performance in the use process [17–19]. The main component of the regenerant is a low-viscosity oil material. Rejuvenators can improve the viscosity of aged asphalt by adjusting the proportion of light-to-heavy components in the asphalt, which improves its road performance and achieves the regeneration effect. Relevant studies have shown that the main components of vegetable oil waste are very similar to those of petroleum asphalt in their elemental composition, and vegetable oil waste has the characteristics of soft, low viscosity, and a high content of light components. Therefore, it can be used as a renewable, economical, and environmentally friendly regenerant [20]. Polyethylene (PE) materials are widely used in daily life, but the treatment of recycled PE is quite complicated. Using recycled PE and R-oil to modify recycled asphalt not only increases the ductility and toughness of the recycled asphalt, but also reduces white pollution [21–25].

In 2014, Chen Meizhu et al. [26] studied the feasibility of vegetable waste oil and cottonseed oil as aged asphalt regenerants. The study found that compared with traditional regenerants, a small amount of vegetable oil waste and cottonseed oil can enhance the fatigue performance of aged asphalt, but the ability to resist high-temperature rutting decreases slightly. In 2016, Gong et al. [27] used biodiesel waste to prepare a regenerant. Their research showed that bio-oil can significantly improve the workability of aged asphalt, but the moisture-proof performance of bio-oil recycled asphalt needs to be further improved. In 2018, Cao Xinxin et al. [28] studied the influence of vegetable waste oil on the pavement performance of aged asphalt. The results showed that when the content of bio-oil reaches 15%, the road performance of rejuvenated asphalt is consistent with that of virgin asphalt, except the high temperature rutting resistance. In addition, waste PE has also been used for many years to modify asphalt. It was found that rubber and PE composite-modified asphalt can also improve the high-temperature performance of asphalt, and greatly improve the temperature sensitivity of composite-modified asphalt. In 2016, Han Jun [29] studied the influence of the content of PE and Crumb Rubber (CR) on the mechanical properties of asphalt, but the research on the influence of PE and CR content on the rheological properties of asphalt is not detailed; In 2017, Chen Changxin [30] and others studied the composition ratio of CR/PE-modified asphalt and its corresponding mechanical properties but did not analyze its storage stability.

In conclusion, bio-oil can improve the low-temperature crack resistance and fatigue performance of aged asphalt, but it reduces the high-temperature rutting resistance, whereas PE can improve the high-temperature performance of asphalt. Currently, researchers mainly pay attention to the application of bio-oil on the rejuvenating aged asphalt. However, there are few studies on modification of bio-oil rejuvenated asphalt. Therefore, this study considered the application of vegetable oil waste in the regeneration of aged asphalt and the use of recycled PE to modify recycled asphalt, which gives a way to prepare environmentally friendly, recycled modified asphalt.

## Objective and experimental plan

The purpose of this study was to obtain an environment-friendly recycled asphalt binder by using R-oil, recycled polyethylene and aged asphalt. The road performance of the material was studied by physical property test, rheological property test and chemical composition analysis. It aims to provide an environment-friendly treatment for R-oil, asphalt recovery and polyethylene recovery.

First, aged asphalt was prepared by an aging treatment: a rotating-film oven test (RTFOT) and a pressurized aging vessel (PAV) were carried out to simulate the aging process of asphalt in the process of heating, mixing, transportation, and use.

Then, the aged asphalt was divided into two test groups, A and B, to which 5%, 10%, 15%, and 20% R-oil were added. 2% recycled PE particles were added to group A, and 4% recycled PE particles were added to group B. Eight kinds of recycled, modified asphalt samples were obtained, among which low-density PE (LDPE) was used as recycled PE particles.

Next, a conventional physical index test, a rheological test, and a chemical composition analysis test were used to compare and study the road performance of eight kinds of asphalt test samples.

The conventional physical index tests included a penetration test, a softening point test, and a ductility test.

Compared with conventional test indices, rheological test indices have a better correlation with the road performance of asphalt binder. The rheological test included a viscosity, a multiple-stress creep, a time scanning test, and a bending beam rheological test.

**Fig 1:**
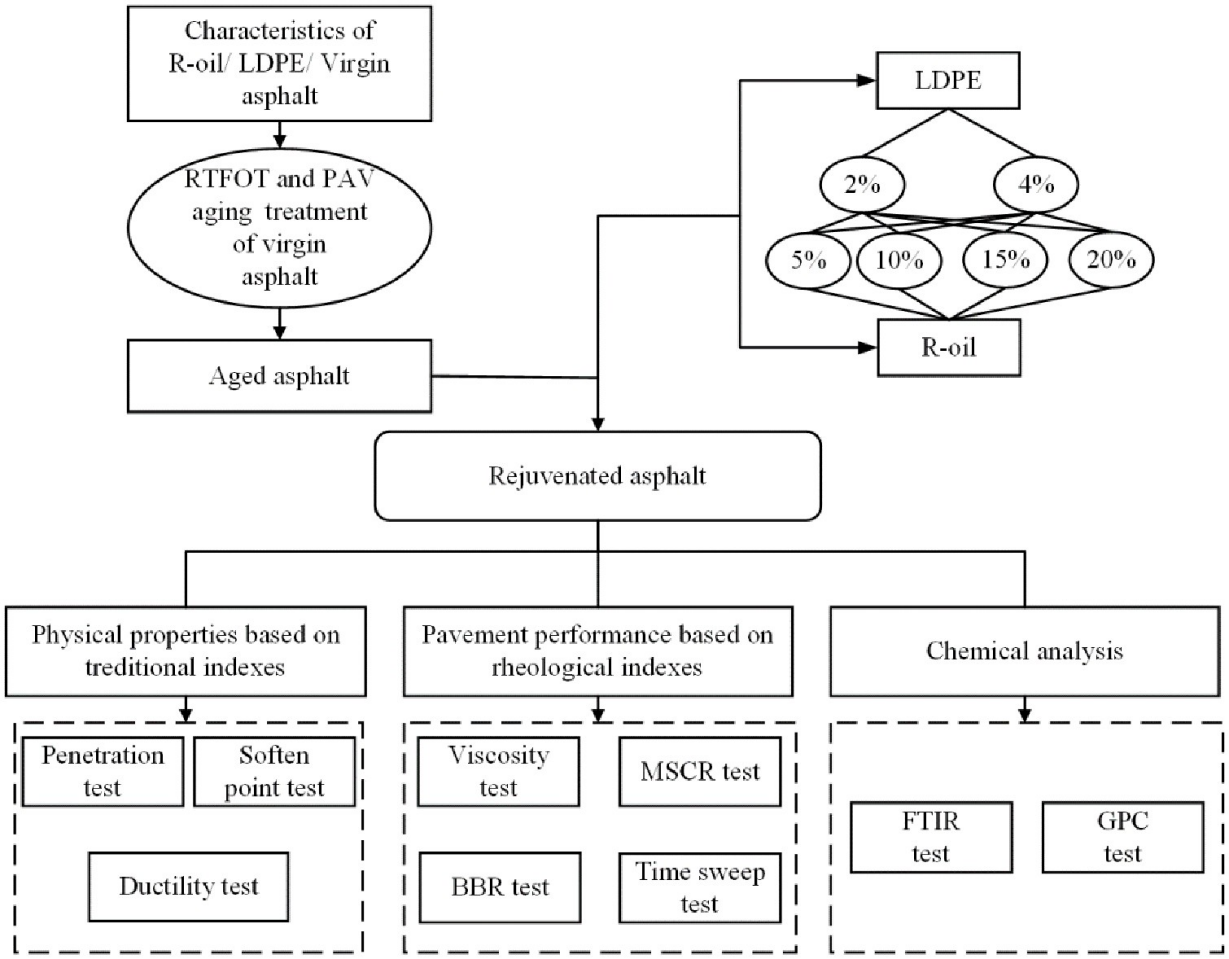
Experimental plan.

Chemical tests included infrared spectroscopy and gel chromatography analysis. By measuring the composition of the functional groups and their molecular weights, the differences in chemical characteristics between recycled and matrix asphalt would be studied, and the correlation between their chemical and rheological properties would be analyzed. From a microscopic perspective, the performance of the recycled vegetable oil waste and recycled PE-modified asphalt would be studied. The experimental plan is shown in Fig 1.

## Materials and testing methods

### Materials

#### R-oil

**Fig 2:**
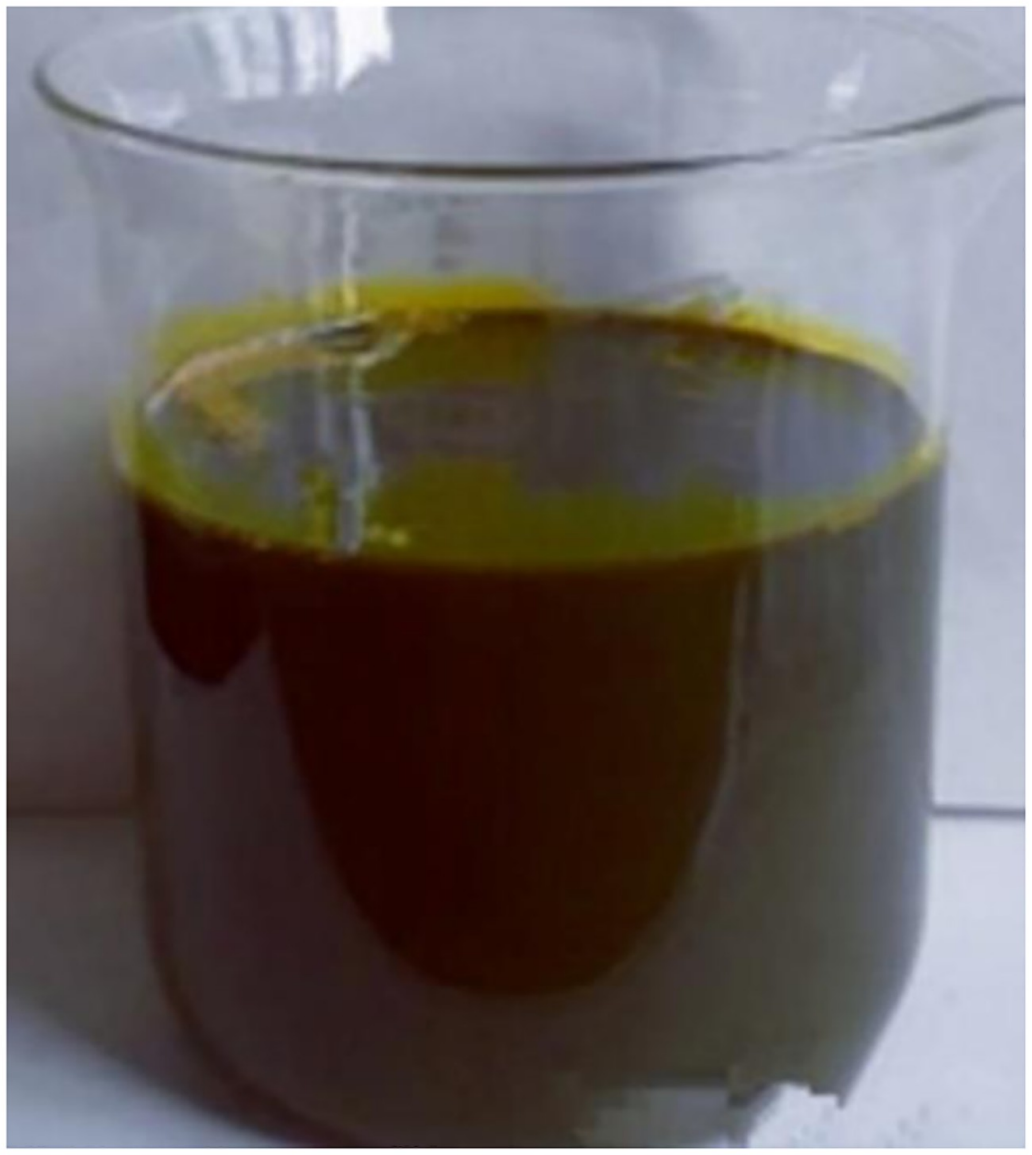
R-oil.

About 15% vegetable oil waste is produced in the process of distillation after acidification. The production of recycled vegetable oil waste (R-oil) is huge in the world, as shown in Fig 2. It is usually treated by burning, which produces toxic and greenhouse gases. This is a waste of natural resources. The physical indices of R-oil are shown in Table 1.

**Table 1:** Physical and chemical properties of R-oil.

Physical and Chemical Properties	Color	Density (g/cm^3^)	Flash Point (°C)	RTFOT Quality Loss (%)	Carbon Content (%)	Hydrogen Content (%)	Viscosity at 60 °C (mPa·s)
	Brown	0.970	281	0.8	78.10	10.29	287.3

#### Recycled PE particles

**Fig 3:**
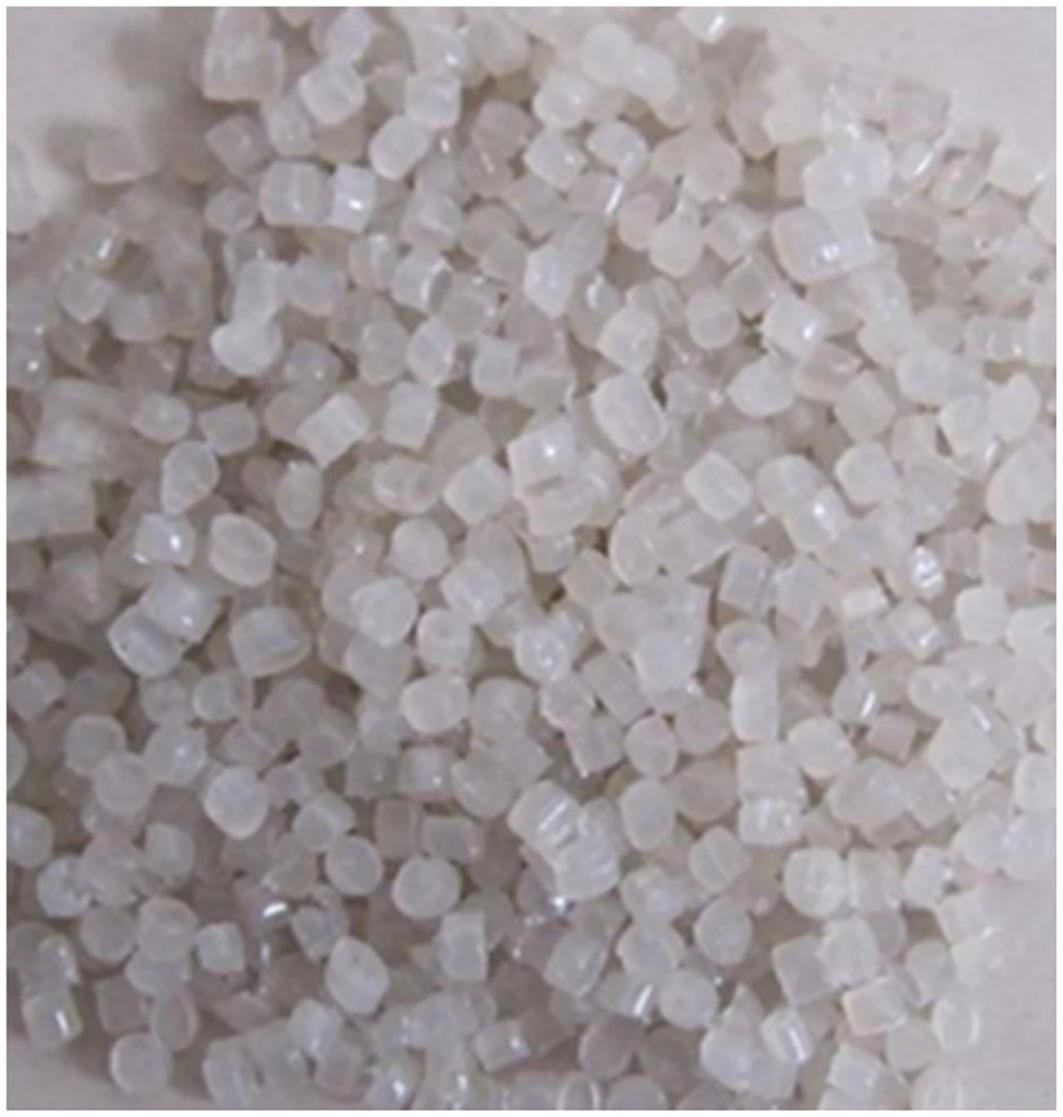
Low-density PE (LDPE) particles.

Low-density PE (LDPE) was selected as the recycled PE particles. The properties of LDPE are shown in Table 2. Some LDPE particles are shown in Fig 3.

**Table 2:** Physical and chemical properties of LDPE.

Physical and Chemical Properties	Density (g/cm^3^)	Softening Point (°C)	Melting Point (°C)	Tensile Strength (mPa)	Melt Flow Rate (g/10 min)
	0.920	92	108	11	50

#### Virgin and aged asphalt

SK70 virgin asphalt from Korea was selected. Aged asphalt was prepared by the short- and long-term aging treatments of SK70 matrix asphalt. The short-term aging mainly simulated the aging of asphalt through a process of heating, transportation, and mixing. The test method referred to ASTM D 2872 [31], and the short-term aging process of asphalt was simulated by a rotating-film oven test. The long-term aging mainly simulated the aging of asphalt binder after 6–8 years in asphalt pavement. The test method referred to ASTM D 6521 [32], and the long-term aging process of asphalt was simulated using a pressure-aging vessel (PAV). The technical indices of the virgin and aged asphalt are summarized in Table 3. The workability and fatigue properties of virgin and aged asphalt are summarized in Table 4. The high and low temperature rheological properties of virgin and aged asphalt are summarized in Table 5.

**Table 3:** Technical specifications of virgin and aged asphalt.

Index	Softening Point (°C)	Ductility (5 °C/cm)	Penetration (25 °C, 5 s/0.1 mm)
Virgin asphalt	52.3	10.5	67.3
Aged asphalt	67.1	0.7	27.8

**Table 4:** Workability and fatigue properties of virgin and aged asphalt.

Index	Mixing temperature (°C)	Compacting temperature (°C)	Fatigue index N_f50_ (cycles)
Virgin asphalt	160	142	67.3
Aged asphalt	170	183	27.8

**Table 5:** High and low temperature rheological properties of virgin and aged asphalt.

Index	Deformation recovery rate under 0.1 kPa/3.2 kPa (%)	Nonrecoverable creep compliance under 0.1 kPa/3.2 kPa (kPa^-1^)	Creep stiffness (mPa)	Creep rate
Virgin asphalt	5.94/1.55	0.26/0.32	67.3	0.5
Aged asphalt	5.68/1	0.58/0.65	27.8	0.1

#### Rejuvenated asphalt

The preparation process of recycled vegetable oil waste and recycled PE regenerated asphalt was determined as follows: R-oil and aged asphalt were heated to 135 °C, respectively. R-oil was mixed into the aged asphalt, and a high-speed shear machine would share the mixture. The shear rate was 3,000 r/min, and the shear temperature was maintained at 135 °C for 30 min. Then, the recycled asphalt was heated to 170 °C and LDPE particles and a cracking agent were added in fractions. No obvious solid particles were found in the asphalt before each addition. The temperature was then raised to 180–185 °C and maintained, plasticizer and solubilizer were added, and the high-speed shear equipment was started to pre-shear at 3,000 r/min for 20 min. The rotational speed was then increased to 6,000 r/min for 100 min, and after shearing the asphalt was cut. After being placed in the oven at 130 °C for two hours for healing, no bubbles appeared on the surface of the material. In addition, the test is conducted after the sample is left for 24h. This process can also ensure the healing of asphalt. The contents of R-oil and LDPE are shown in Table 6. The technical specifications of solubilizer and plasticizer are shown in Tables 7 and 8. As shown, eight kinds of recycled asphalt binders were prepared.

**Table 6:** Proportion of formula of rejuvenated asphalt.

Sample code	1	2	3	4
A	2% LDPE + 5% R-oil	2% LDPE + 10% R-oil	2% LDPE + 15% R-oil	2% LDPE + 20% R-oil
B	4% LDPE + 5% R-oil	4% LDPE + 10% R-oil	4% LDPE + 15% R-oil	4% LDPE + 20% R-oil

**Table 7:** Technical specifications of solubilizer.

Appearance	Flash Point (°C)	Viscosity at 100 °C (mPa·s)	pour point (°C)
Dark brown liquid	230	35	18

**Table 8:** Technical specifications of plasticizer.

Appearance	Density (g/cm^3^)	Flash Point (°C)
Colorless liquid	1.05	200

### Testing methods

#### Physical tests

**Fig 4:**
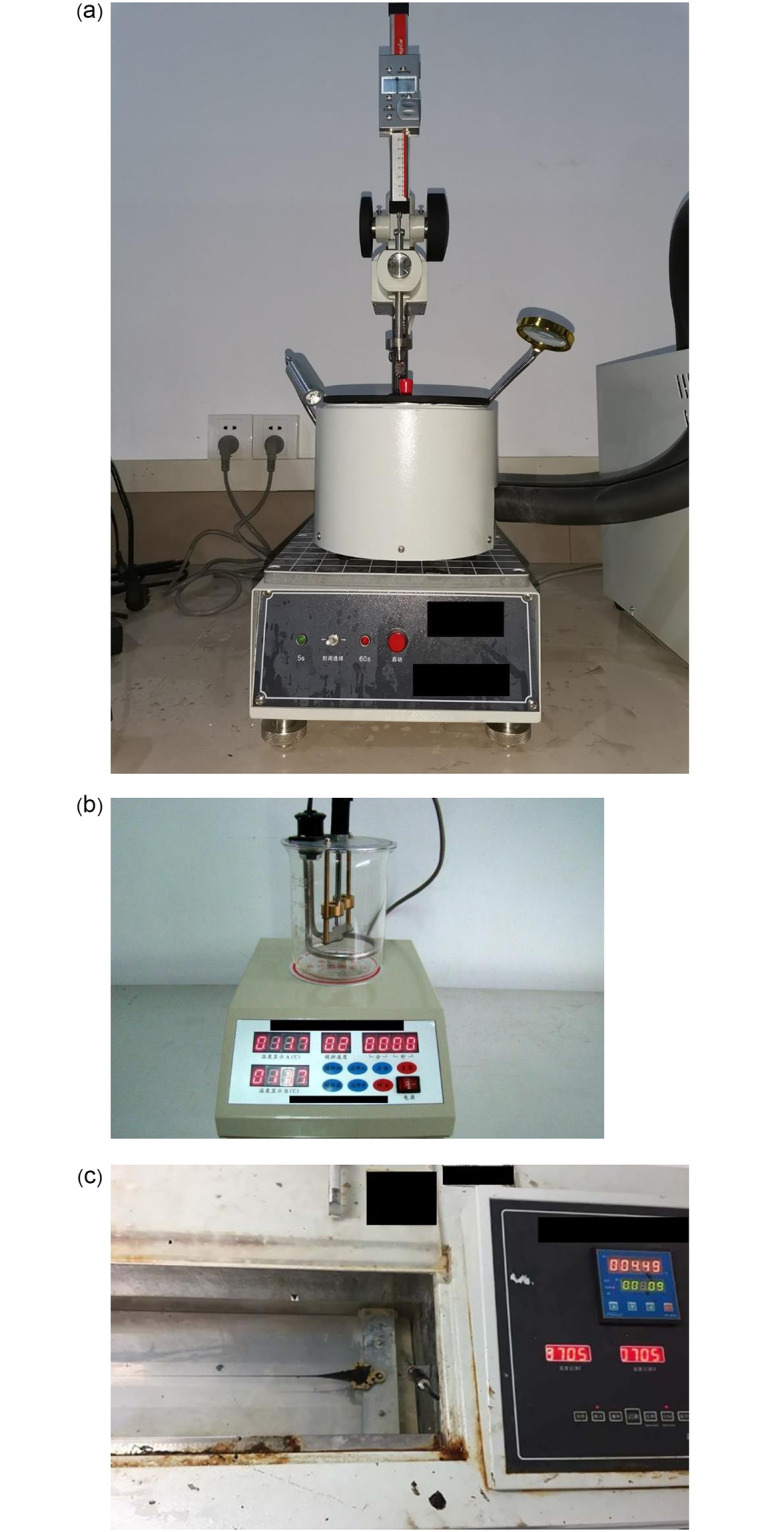
Physical tests: (a) The SYD-2801F Needle Penetration Tester (b) The SLR -V Softening Point Tester (c)The SYD-4508C Ductility Testing Machine.

The conventional physical properties of eight kinds of recycled asphalt were tested, as shown in Fig 4.

As shown in Fig 4, the penetration index is a basis for asphalt classification. The specific test method referred to ASTM D5 [33]. The softening point test is a method for evaluating the high-temperature performance of asphalt. The specific test method referred to ASTM D36 [34]. The ductility index is a reflection of the ductility of the asphalt. The specific test method referred to ASTM D113 [35].

#### Rheological tests

(1) Viscosity test

The high-temperature viscosity of asphalt can characterize its construction and workability. A Brookfield viscometer was used to test the viscosity of asphalt at 120 °C, 135 °C, 150 °C, 165 °C, and 180 °C. The specific test method referred to ASTM D4402 [36].

(2) Multiple-stress creep recovery (MSCR) test

At present, the MSCR test has been proven to effectively evaluate and distinguish the ability of various types of modified asphalt to resist permanent deformation, and its results have a good correlation with the asphalt mixture rutting test [37–39]. Vegetable waste oil and recycled PE reclaimed asphalt has obvious differences in physical and chemical properties compared with base asphalt, and can be considered as a modified asphalt binder. In order to more accurately predict the road performance of vegetable waste oil and PE regenerated asphalt under high-temperature conditions, an MSCR test was performed on eight samples, and the irrecoverable compliance J_nr_ and deformation recovery rate R were selected as evaluation indices.

The MSCR test used an AR 2000 dynamic shear rheometer (DSR). Two creep stress levels of 0.1 and 3.2 kPa were set, and the test temperature was set to 60°C, testing the ability of the sample to resist deformation and high temperatures under light and heavy load conditions. Each stress level was continuously tested for 10 cycles within a total duration of 200 s. Each cycle was divided into a 1s creep phase and a 9 s unloading recovery phase.

(3) Time sweep test

The time scan test results can well reflect the fatigue damage characteristics of asphalt binders and their ability to resist the repeated actions of loads. They also have a strong correlation with the fatigue performance of asphalt mixtures.

The DSR was set to the 5% strain control mode, the diameter of rotor was 25 mm, the distance between parallel plates was 1 mm, the test temperature was 20 °C, the scanning frequency was 10 rad/s, and the fatigue performance evaluation index was N_f50_, while the complex modulus was reduced to 50%.

(4) Bending beam rheometer test

Low-temperature bending rheological tests (BBR) were carried out using a TE bending beam rheometer. Creep stiffness S and stiffness change rate m were selected as the evaluation indices of asphalt binder cracking at low temperature. Based on the time–temperature equivalent principle, the corresponding load and deformation values at 60 s were selected, and the test temperature was set to –24 °C, –18 °C and –12 °C.

#### Chemical tests

(1) Fourier transform infrared spectrometer (FTIR) test

**Fig 5:**
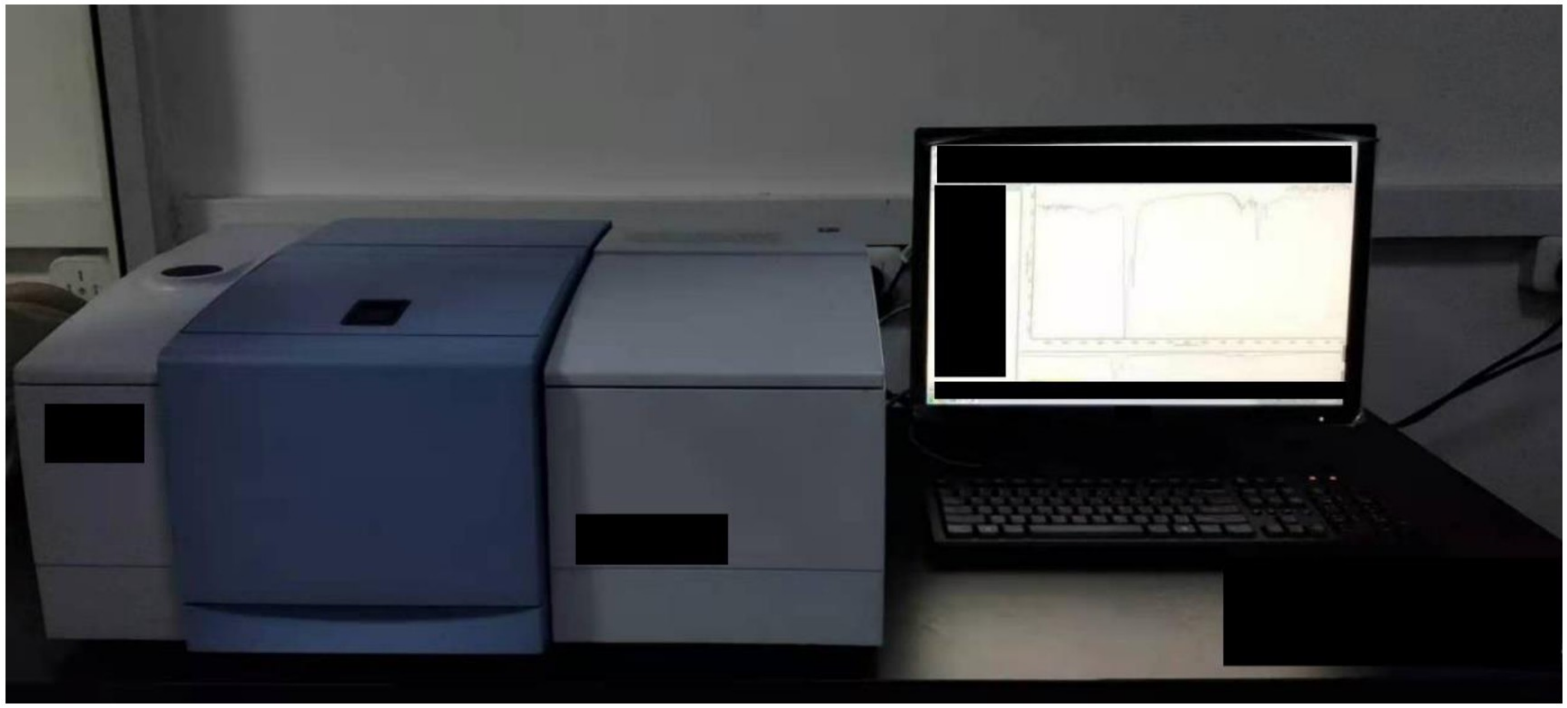
Fourier transform infrared spectrometer.

The model of the infrared spectrometer was Nicolet740 FTIR. The resolution was 4 cm^–1^, the number of scans was 32, and the measuring range was 4000–400 cm^–1^. The sample was prepared by the solution method. It was dissolved in the organic solvent trichloroethylene and then tested, as shown in Fig 5.

(2) Gel permeation chromatography (GPC) test

The model of the infrared spectrometer was TOSOH HLC-8320GPC.GPC was carried out for matrix asphalt, aging bitumen, and B3 samples, and gel chromatograms were processed. According to the method of molecular classification [40], the asphalt constituents were divided into small-sized molecules (SMS), medium-sized molecules (MMS), and large-size molecules (LMS). The gel permeation chromatograms of three kinds of bitumen were compared and analyzed.

## Results and discussion

### Pavement performance based on physical tests

#### Penetration

**Fig 6:**
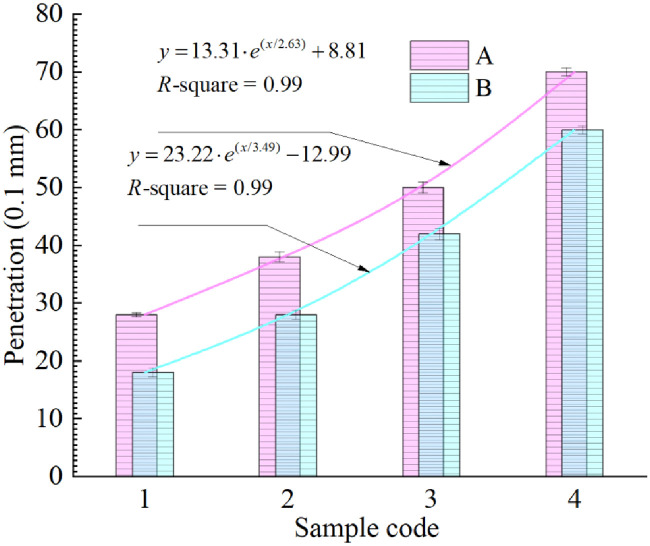
Penetration of eight rejuvenated asphalt samples.

The higher the penetration index is, the softer the asphalt is and the lower its viscosity is. It can be seen from Fig 6 that under the same R-oil content, the penetration index of group A is higher than that of group B, indicating that an increase in LDPE content can reduce the penetration of samples. In addition, with an increase in R-oil content, the penetration values of groups A and B show strong exponential growth. The penetration value of sample A4 is 0.27 mm lager than that of virgin asphalt, while that the value of A1 is 3.93mm less than that of virgin asphalt. This shows that R-oil can effectively enhance the penetration of aged asphalt.

#### Softening point

**Fig 7:**
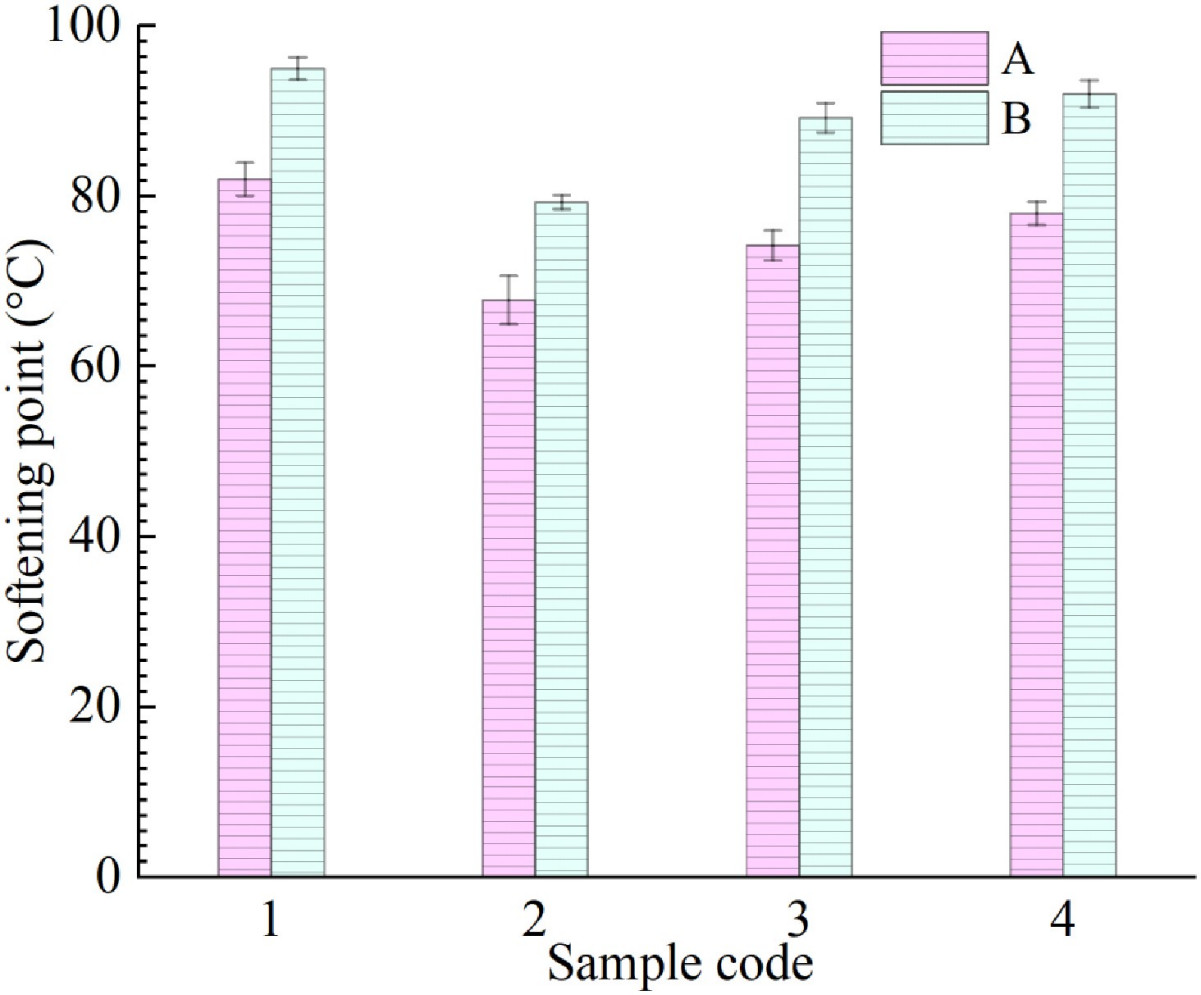
Softening point of eight rejuvenated asphalt samples.

The softening point index is an important index for characterizing the thermal stability of asphalt binder, and it has a positive correlation with the thermal stability of asphalt. Fig 7 shows that the softening point indices of the group A and group B samples show an obvious two-stage change trend. In practical engineering application, with the increase of asphalt pavement service time, the light components in asphalt binder volatilize, which leads to the increase of softening point of aged asphalt. With an increase in R-oil content, the softening point first decreases and then increases. This indicates that LDPE cannot effectively improve the thermal stability of aged asphalt when the R-oil content is small (i.e., the aged asphalt is not fully regenerated). However, after the aged asphalt is rejuvenated, LDPE can significantly improve its thermal stability.

#### Ductility

**Fig 8:**
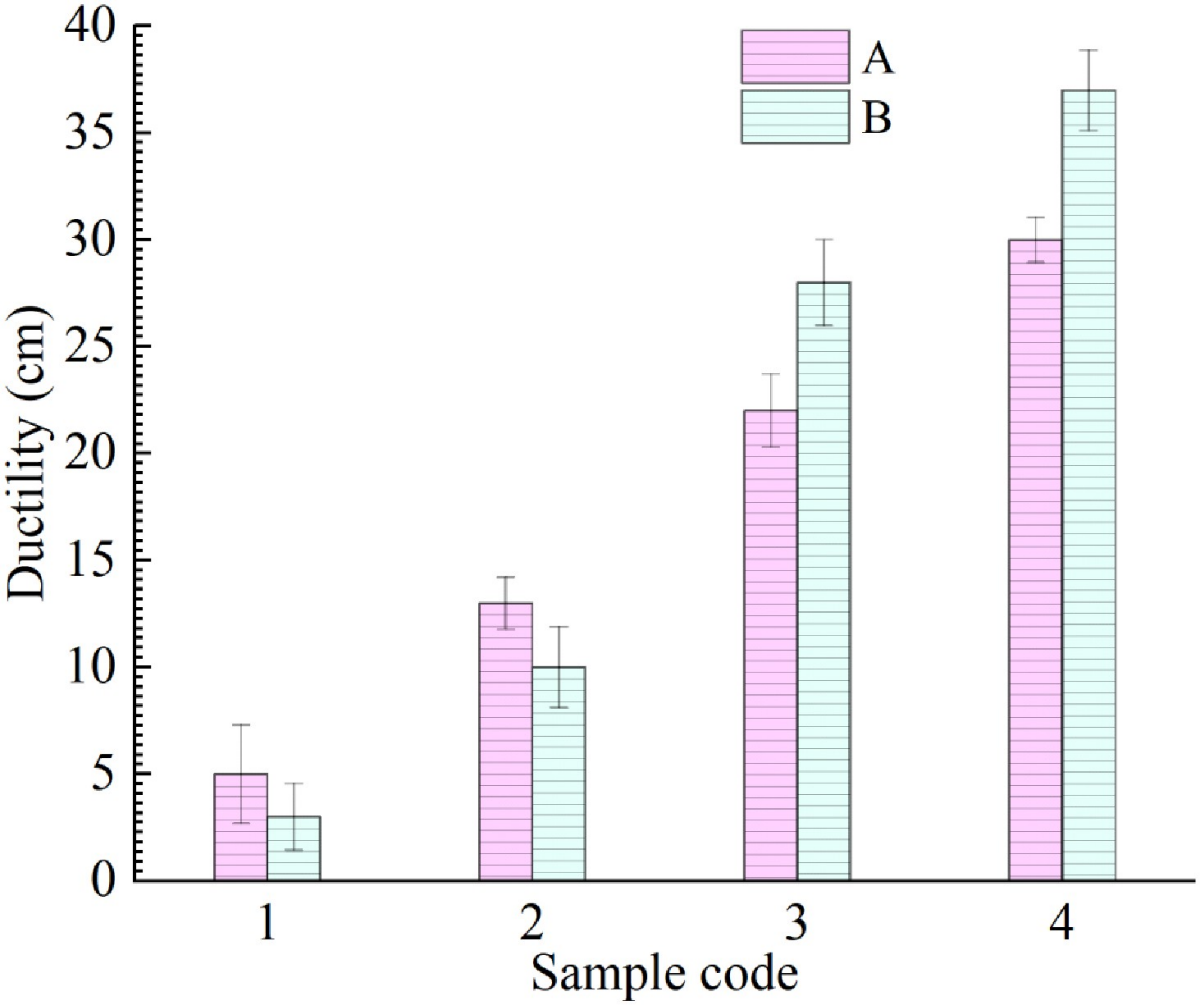
Ductility of eight rejuvenated asphalt samples.

Ductility reflects the ductility of asphalt. The higher the ductility, the better performance the asphalt has. The results show that the ductility of A4 is 19.5 cm longer than that of virgin and that the ductility of B4 is 26.5 cm greater than that of B2, which implies that R-oil improves the ductility of aged asphalt. The test results show that an increase in LDPE content is unfavorable for the low-temperature ductility of aged asphalt when the R-oil content is no more than 10%. The Fig 8 shows that the ductility of A4, A3 is lower than that of B4, B3, respectively. It suggests that when the R-oil content exceeds 10%, LDPE can improve the low-temperature ductility of rejuvenated asphalt.

### Pavement performance based on rheological tests

#### Workability evaluation

**Fig 9:**
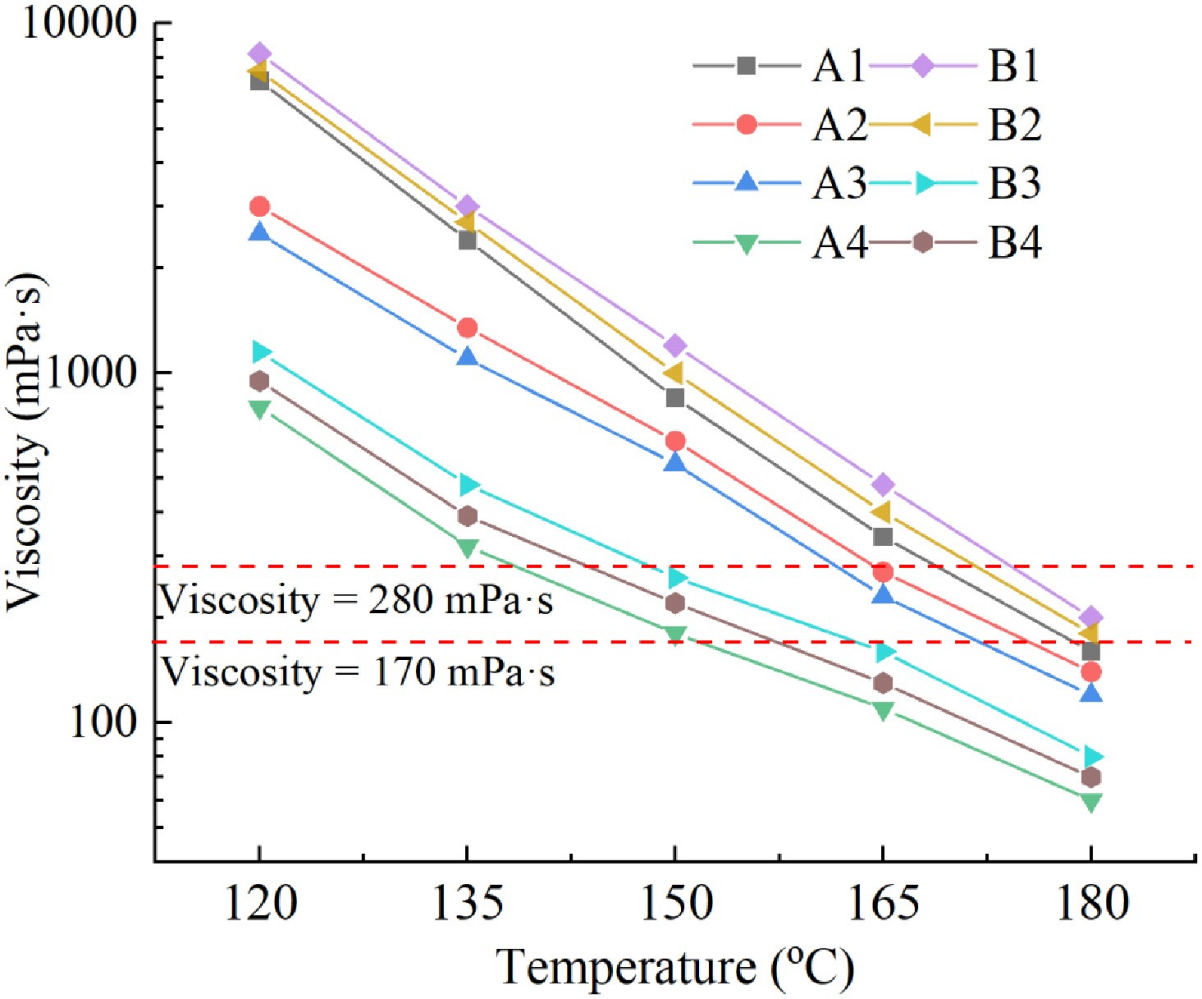
Viscosity–temperature curve of eight rejuvenated asphalt samples.

Viscosity is an important technical index for characterizing asphalt performance, and a reasonable viscosity range is the guarantee of asphalt mixture performance. The "Highway Asphalt Pavement Construction Technical Specification" (JTG F40-2004) recommends that the best mixing viscosity for asphalt binder is 170 mPa·s and the best compaction viscosity is 280 mPa·s. Fig 9 shows the viscosity–temperature curves of eight kinds of asphalt samples at 120 °C to 180 °C. It can be seen from these results that the higher the test temperature is, the lower the viscosity of the asphalt is. Therefore, the test temperature that corresponds to the optimal viscosity can be found from the viscosity–temperature curve to determine the optimal mixing and compacting temperatures. In practical engineering applications, the lower the construction temperature is, the less energy is needed for heating, and the more economical and environmental friendly the production process is. When the test temperature is fixed, the viscosity of vegetable oil waste and recycled PE regenerated asphalt has different degrees of decline after the composite modification with R-oil and LDPE. The best mixing and compacting temperatures of the B4 asphalt sample are 157.5 °C and 144 °C, respectively, and the two indices are close to those of virgin asphalt. The reason for improving the workability of recycled asphalt lies in the dilution function provided by the R-oil component. In addition, Fig 9 shows that when the R-oil content is 5%, 10% or 20%, increasing the LDPE content will increase the viscosity, but when the R-oil content is 15%, the A3 group is obviously larger than the B3 group. The reason for this phenomenon is that the aged asphalt is not completely rejuvenated when the R-oil content is less than 15%, and the modification effect of LDPE on the rejuvenated asphalt is poor. The aged asphalt is completely rejuvenated when the R-oil content reaches 15%, the modification effect of LDPE on the rejuvenated asphalt is the best. When the content of LDPE is increased, the viscosity of asphalt decreases, which shows that the viscosity of B3 group is lower than that of A3 group and it also indicates that B3 group has better workability. When the content of R-oil reaches 20%, there will be some excessive R-oil, which leads to the poor combination of LDPE and rejuvenated asphalt. The increase of LDPE content will enhance the viscosity of asphalt.

**Fig 10:**
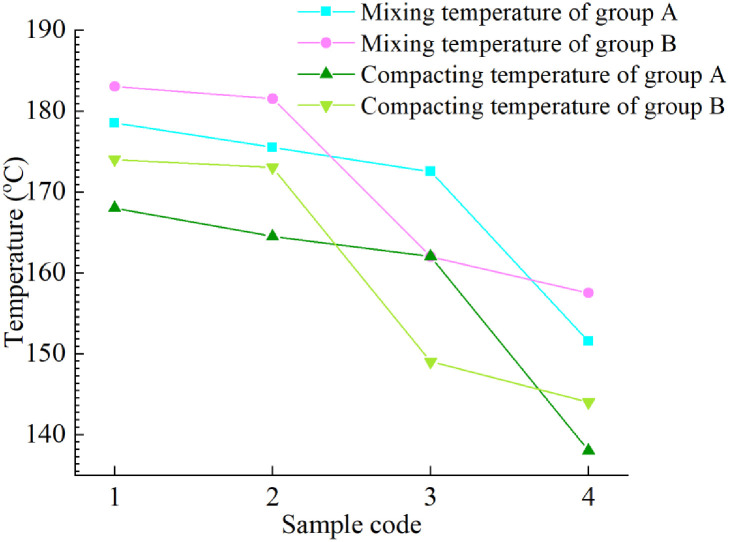
Mixing and compacting temperature of eight rejuvenated asphalt samples.

Fig 10 shows the best mixing and compacting temperatures of groups A and B asphalt samples. From these results, when the content of LDPE is constant, with an increase in the R-oil content, the optimal mixing and compacting temperatures of groups A and B continuously decrease. At the same time, it can be seen that for the group A samples, when the R-oil content reaches 20%, the best mixing and compaction temperatures of A4 are 21 °C and 24 °C lower than those of A3, respectively; for group B, when the R-oil content reaches 15%, the best mixing and compaction temperatures of B3 are 19.5°C and 24°C lower than those of B2, respectively. It indicates that the temperature turning points of group A and group B are different.

#### Pavement performance under high temperature conditions

**Fig 11:**
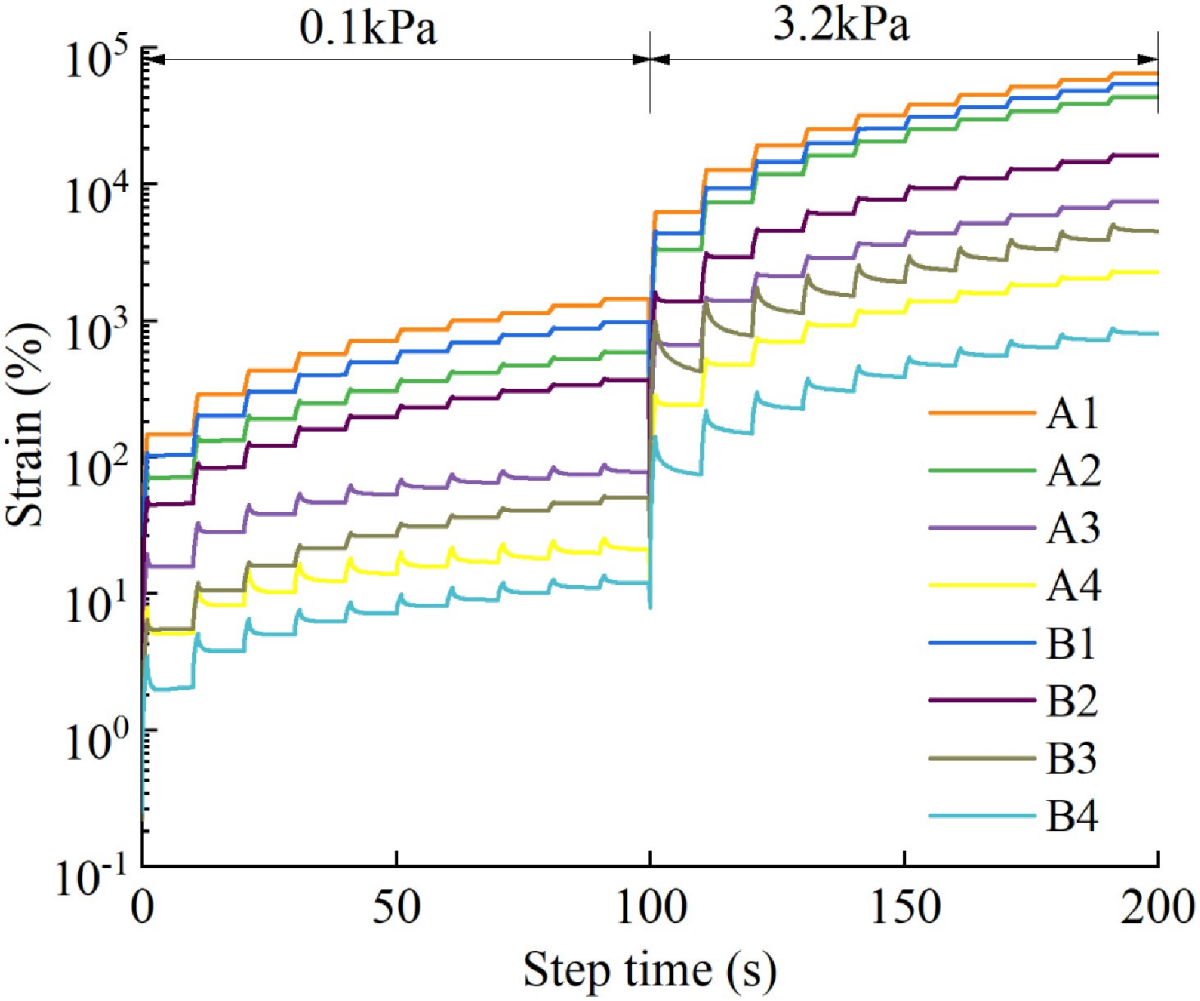
Time–strain response of eight kinds of asphalt binders in MSCR test at 60 °C.

The MSCR test results are summarized in Fig 11. It can be seen from these results that the distinction between the various creep recovery curves is obvious. The creep deformation of the A4 asphalt binder is significantly lower than that of the A1 binder and higher than that of the B4 binder, indicating that both R-oil and LDPE can significantly heighten the high-temperature rutting resistance of the asphalt. Under a high temperature and light load (0.1 kPa), when the content of the asphalt binder modifier is changed from B2 to A3, the strain level drops significantly, whereas under a high temperature and heavy load (3.2 kPa), the strain level decline is relatively even when the content of the modifier changes.

**Fig 12:**
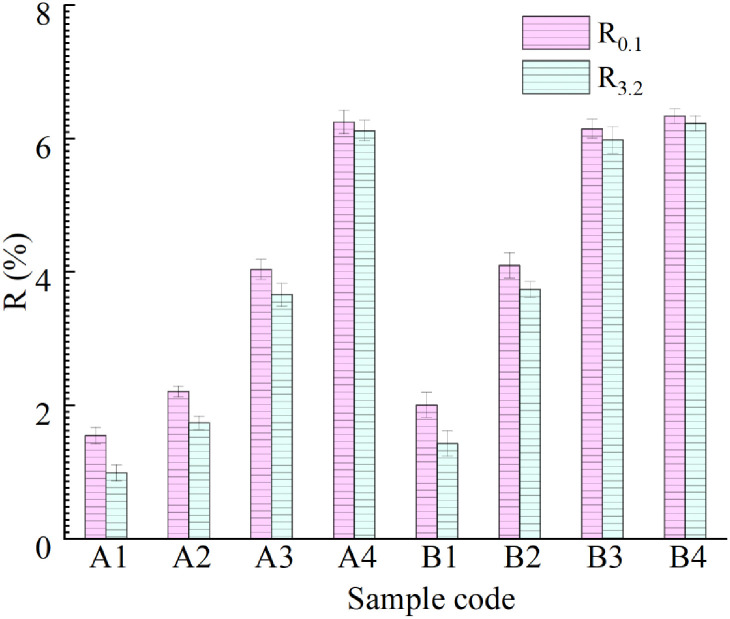
Comparison of creep recovery rate R of eight kinds of asphalt binders.

The MSCR test data for eight kinds of asphalt binder were calculated, and the deformation recovery rate R_0.1_, R_3.2_ and irrecoverable creep compliance J_nr0.1_, J_nr3.2_ of the asphalt binders under high temperature/light load (0.1 kPa) and high temperature/heavy load (3.2 kPa) were obtained, as shown in Fig 12. It can be seen that the creep recovery rate of each asphalt binder under a stress level of 3.2 kPa is lower than that under a stress level of 0.1 kPa, which is consistent with an actual road use situation. After adding different proportions of R-oil and LDPE modifier, the order of the creep recovery rate of the asphalt binder was B4 > A4 > B3 > virgin asphalt>B2 > A3 > A2 > B1 > A1> aged asphalt. The results show that, the amount of R-oil and LDPE modifier jointly determine the creep recovery rate of vegetable oil waste and recycled PE regenerated aged asphalt.

**Fig 13:**
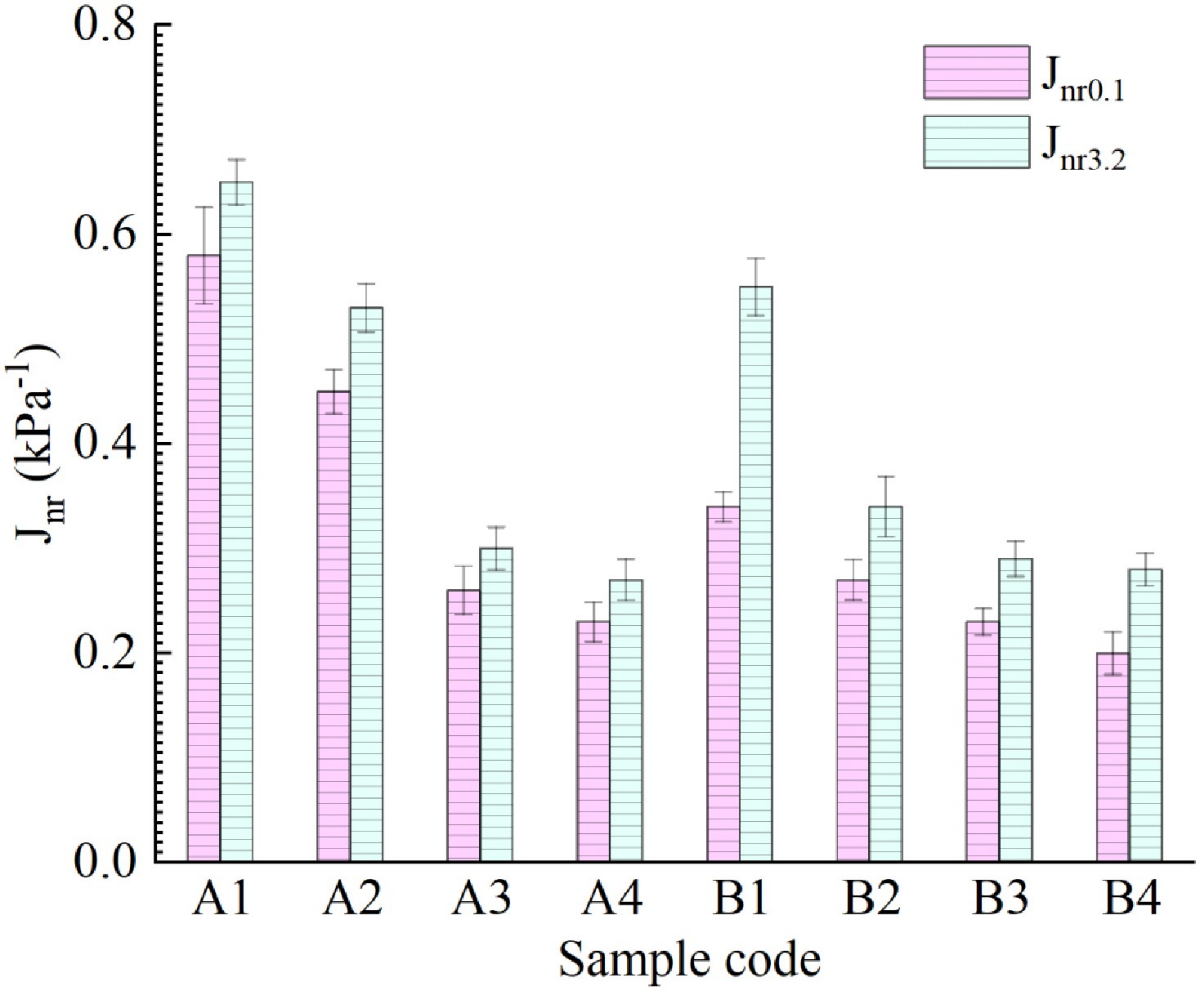
Comparison of nonrecoverable creep flexibility J_nr_ of eight kinds of asphalt binders.

The nonrecoverable creep compliance represents the degree of deformation of asphalt binder under a high-temperature load. The results in Fig 13 show that the resistance to permanent deformation under the 0.1kPa of B4 asphalt binder is the strongest among the eight asphalt binders. Under the same R-oil content, the higher the LDPE content, the smaller the J_nr_ of the asphalt binder. When the content of R-oil and LDPE reaches a certain proportion, the prepared samples A4 and B4 have similar permanent deformation levels. This indicates that the improvement effect of the R-oil and LDPE content on the resistance to permanent deformation of the asphalt binder is no longer obvious after a certain limit is exceeded.

#### Fatigue property

**Fig 14:**
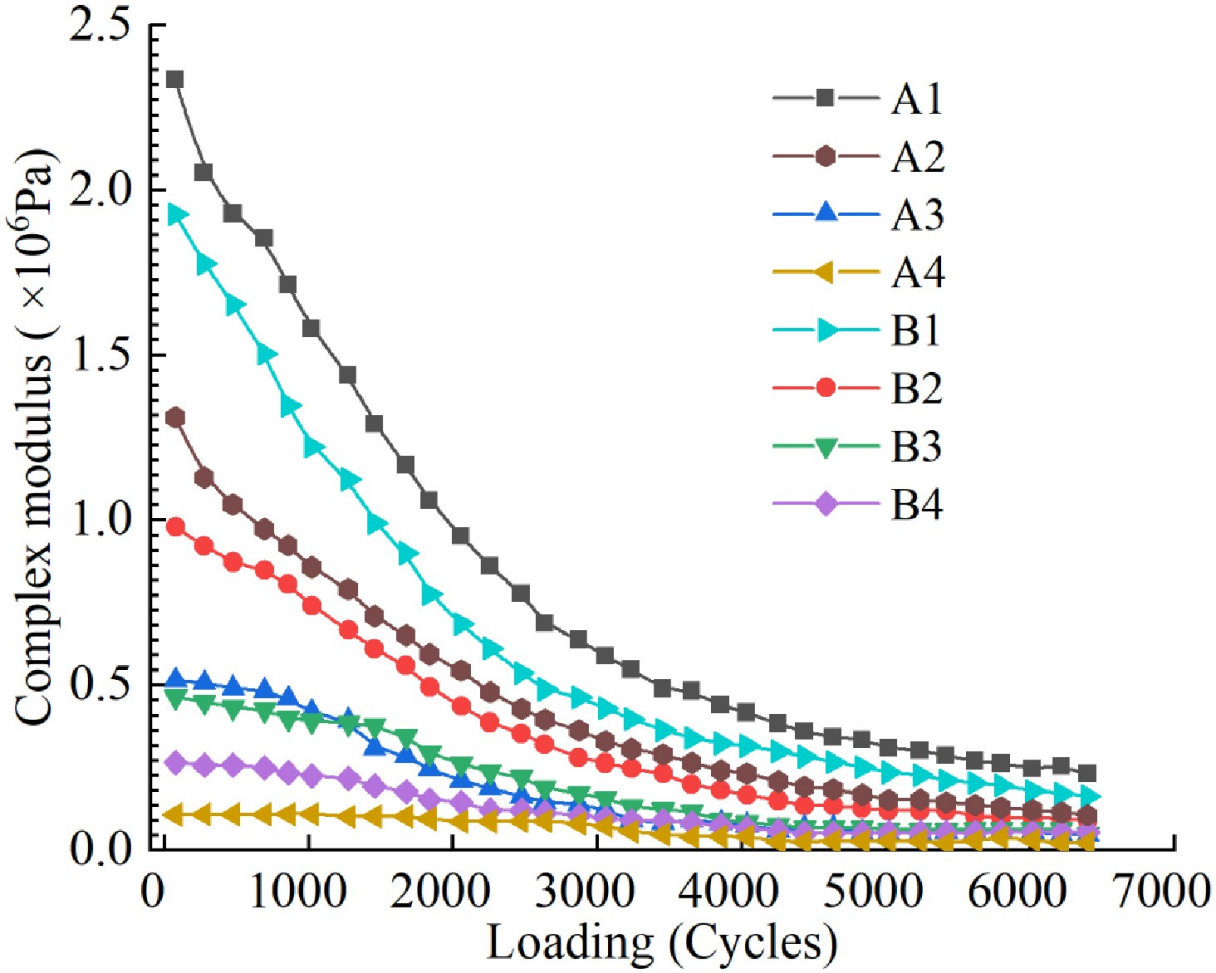
Change of complex modulus of eight kinds of rejuvenated asphalt binders under loading.

In order to more accurately analyze the influence of R-oil and LDPE on the fatigue properties of aged asphalt, the curves of complex modulus of eight kinds of asphalt samples versus the number of loading cycles are shown in Fig 14. It shows that with an increase in loading cycles, the complex modulus of the eight asphalt samples decreases to varying degrees. Also, with an increase in R-oil and LDPE content, the initial complex modulus of the asphalt samples continuously decreases and the declining trend of the complex modulus slows. Among them, the initial complex modulus of A1 is the highest and that of A4 is the lowest. In an actual road use process, the asphalt binder should have a sufficiently high initial modulus while considering fatigue performance. Therefore, the content of R-oil and LDPE should be reasonably controlled according to the rheological test results.

The N_f50_ values of the eight curves were collected to evaluate the fatigue performance index of the regenerated asphalt, as shown in Tables 9 and 10. Comparing the data of groups A and B, it can be seen that R-oil can improve the fatigue life of aged asphalt, and the fatigue life of asphalt has a strong exponential function relationship with the content of R-oil. In addition, under the same R-oil content, the N_f50_ values of group B is greater than that of group A, except the B4 and A4 samples. It indicates that when the R-oil content is more than 15%, the increase of LDPE content has adverse effect on the fatigue life of recycled asphalt.

**Table 9:** N_f50_ of Group A.

Sample code	A1	A2	A3	A4	Fitting curve equation
N_f50_ (cycles)	1587	1672	1836	3454.5	*y* = 0.46·*e*^(*x*/0.48)^ + 1610.85 *R* − *square* = 0.99

**Table 10:** N_f50_ of Group B.

Sample code	B1	B2	B3	B4	Fitting curve equation
N_f50_ (cycles)	1521	1836	2472	2438	*y* = −2191.33·*e*^(−*x*/2.50)^ + 2957 *R* − *square* = 0.74

#### Low-temperature property

**Fig 15:**
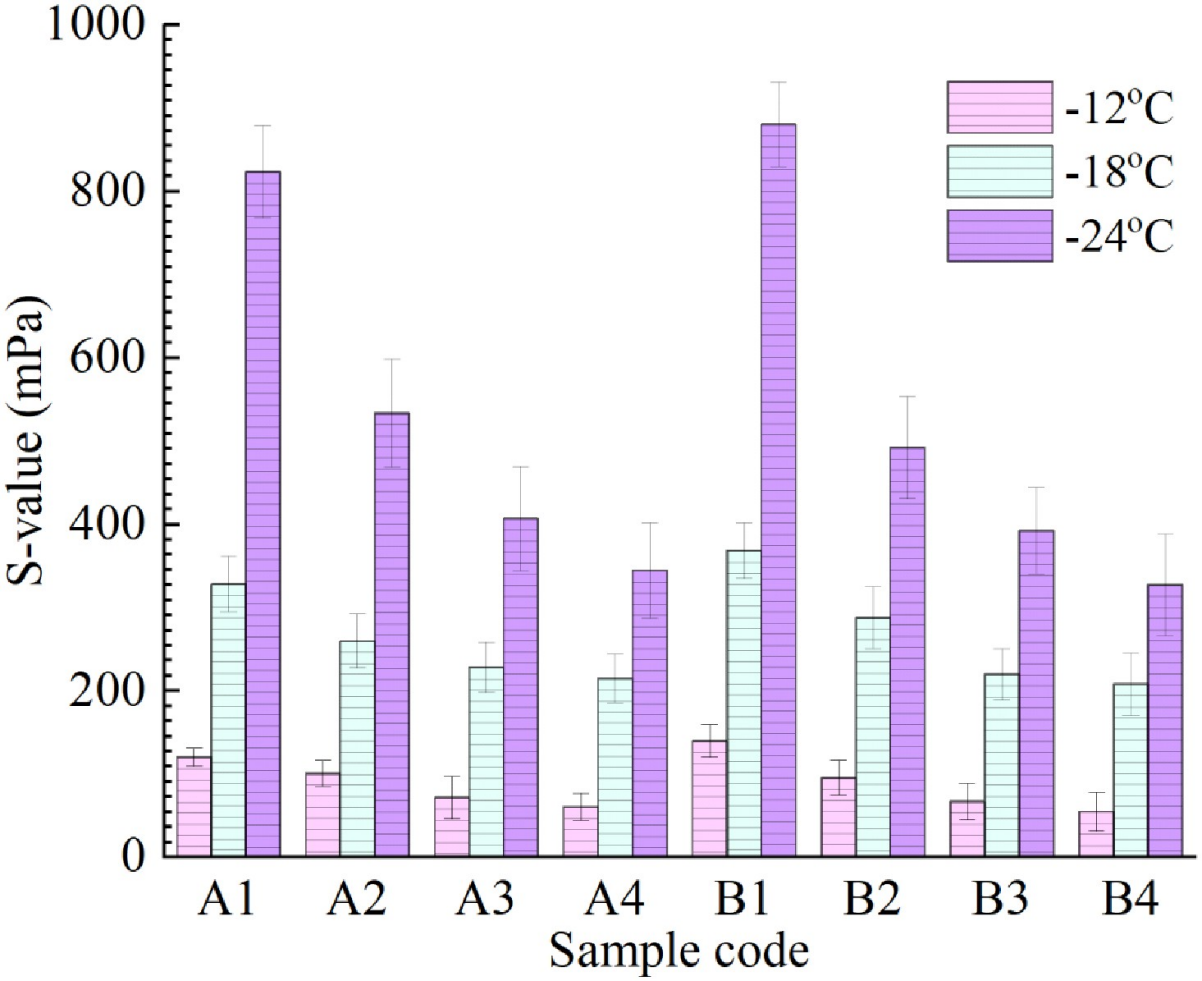
S-values of eight kinds of rejuvenated asphalt.

**Fig 16:**
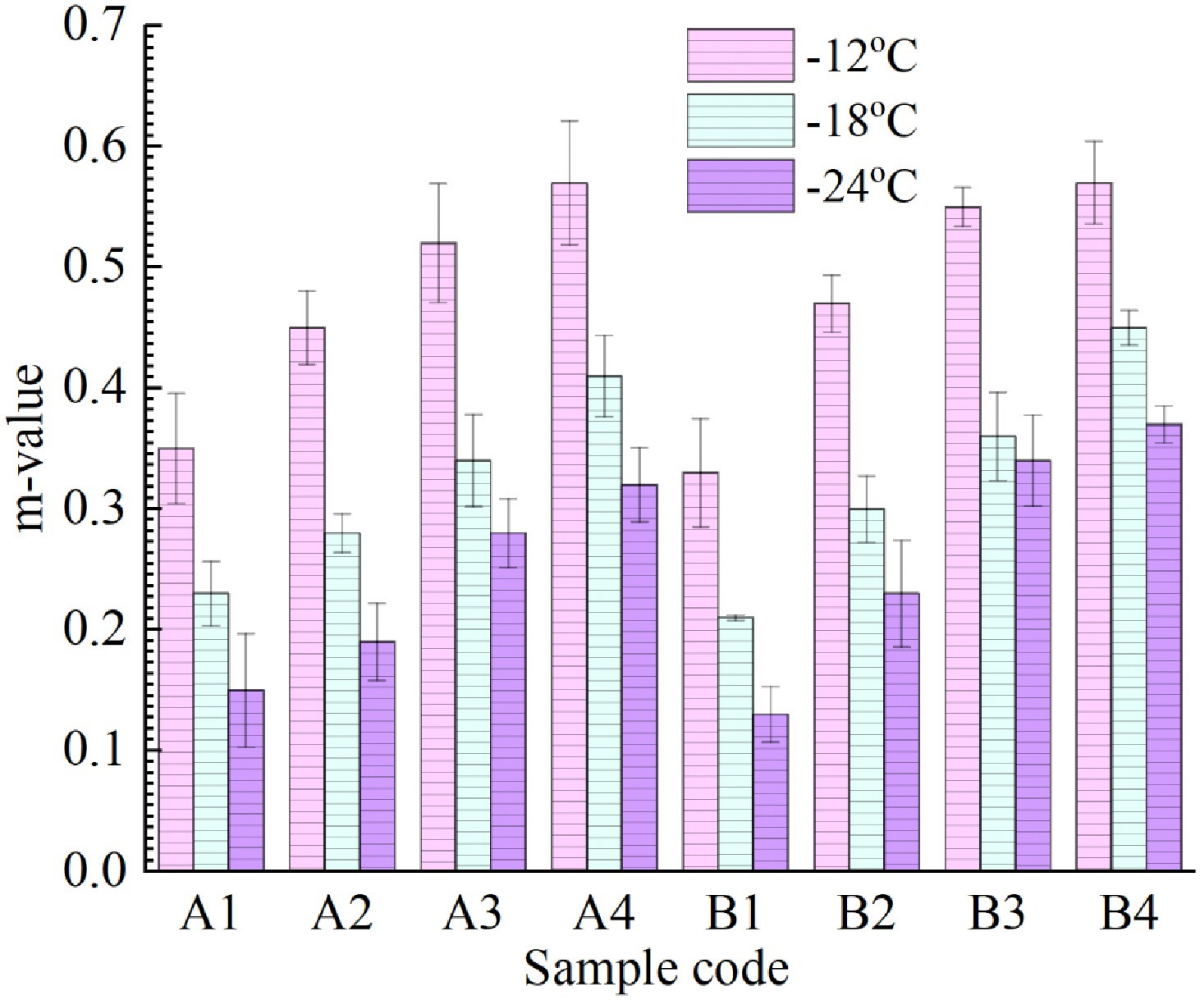
m-values of eight kinds of rejuvenated asphalt.

The results of the creep stiffness modulus S and creep rate m of the eight kinds of asphalt binder at different test temperatures are summarized in Figs 15 and 16. The creep stiffness modulus index S indicates the ability of asphalt to resist permanent deformation under a low-temperature load, and the larger the value is, the worse the low-temperature flexibility is. The creep rate index m indicates the change rate of the creep stiffness modulus of asphalt under a low-temperature load, and the greater the value is, the stronger the stress relaxation of the material is in a low-temperature environment, and the better its low-temperature crack resistance performance is. The following can be seen from the figures: (1) Under the same test temperature and LDPE content, with an increase in R-oil content, the creep stiffness modulus of groups A and B asphalt decreases significantly. This indicates that R-oil can enhance the low-temperature performance of the asphalt. (2) With a decrease in test temperature, the S-value of the eights kinds of asphalt samples gradually decreases, while the m-value gradually increases. This indicates that the lower the low-temperature grade is, the worse the low-temperature flexibility of asphalt binder is, and the stress relaxation performance is decreased. It is easy to produce low-temperature cracking in the actual road use process. The reason for this phenomenon is that asphalt binder is glassy at low temperatures, which is unfavorable to the ductility of asphalt.

### Pavement performance based on chemical tests

#### Infrared spectrum analysis

**Fig 17:**
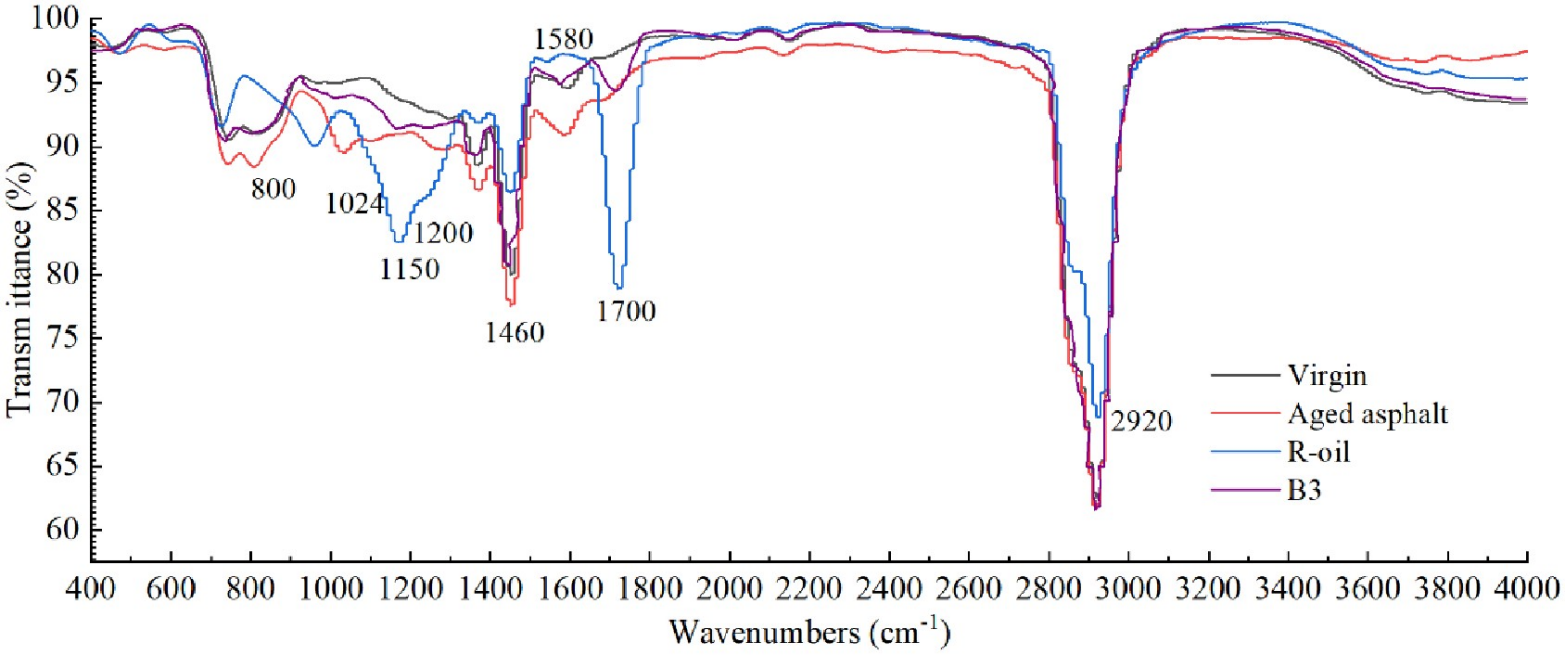
Fourier transform infrared (FTIR) spectra of virgin, aged, B3 asphalt and R-oil.

FTIR can accurately determine the molecular structure and characteristic functional groups of polymers. In order to analyze the reaction of R-oil and LDPE added to aged asphalt and further elaborate its modification mechanism, the infrared spectra of R-oil, base asphalt, aged asphalt, and B3 sample (15% R-oil + 4% LDPE) were tested by FTIR, as shown in Fig 17. The following results can get from the figure: (1) R-oil has strong absorption peaks at 1,150 and 1,700 cm^-1^, but there is no absorption peak at the same position for the virgin asphalt, which indicates that there were many ester bonds in the R-oil sample. (2) There are absorption peaks at 800 and 1,580 cm^-1^ for the base asphalt, but there are no absorption peaks for R-oil at these two positions, which indicates that vegetable oil waste does not contain benzene when used as a regenerant. Nor does it contain carcinogen polycyclic aromatic hydrocarbons; therefore, R-oil can reduce the risk to construction workers involved in the use process. (3) The characteristic absorption peak of the ester group -COOR in R-oil at 1,200 cm^–1^ can be used to characterize the existence of R-oil in the recycled, aged asphalt from vegetable oil waste and recycled PE. In addition, the characteristic peak of carbonyl C = O appears at 1,700 cm^–1^, which indicates that the oxygen absorption reaction of unsaturated carbon chains and elemental sulfur occurred in the preparation process of the B3 sample; (4) At 1,030 cm^–1^, the sulfoxide group is found. There are absorption peaks in the matrix asphalt, aged asphalt, and sample B3, but there is no sulfoxide group absorption peak in the R-oil.

According to the Beer-Lambert law, the content of functional groups is related to the size of the absorption peak and the strength index of the sulfoxide and carbonyl groups can be used to characterize the degree of aging and regeneration of asphalt. Therefore, the carbonyl index (CI), which characterizes the C = O content, and the sulfoxide index (SI), which characterizes the S = O content, are introduced. The calculation results of the three indices are shown in Table 11.

**Table 11:** Carbonyl index (CI) and sulfoxide index (SI) of different asphalt types.

Types of asphalt	CI	SI
Virgin asphalt	0.0230	0.0709
Aged asphalt	0.0449	0.1829
B3	0.1390	0.1098

It can be seen from these results that aging increases the CI and SI indices in asphalt. After adding R-oil and LDPE, the CI index of the aged asphalt increases and the RI index decreases. That is because there is no sulfoxide group in bio-oil, but there is a large amount of the carbonyl group. R-oil dilutes the sulfoxide group with high polarity in the aged asphalt.

#### Gel permeation chromatography (GPC) test

**Fig 18:**
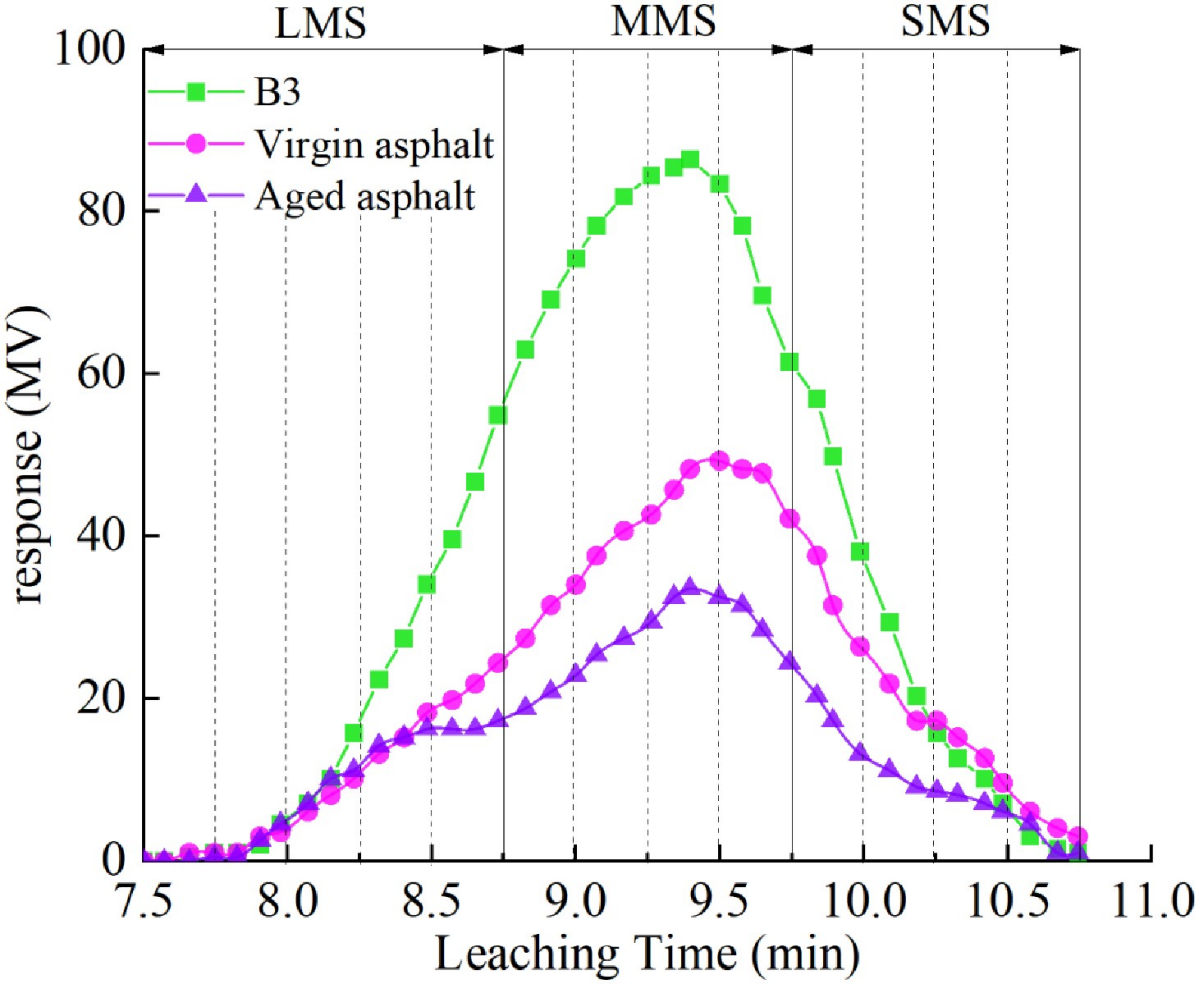
Gel permeation chromatography spectrogram of virgin, aged, and B3 asphalt.

The virgin asphalt, aging bitumen, and sample B3 GPC spectrograms were processed to categorize the molecules as small-sized molecules (SMS), medium-sized molecules (MMS), and large-size molecules (LMS). As shown in Fig 18, the elution time is divided by dotted lines, where the first five sections are LMS, the 6th to 9th sections are MMS, and the 10th to 13th sections are SMS.

The molecular distribution and dispersion of the three kinds of asphalt are shown in Table 12. According to these results, aging increases the content of LMS and decreases the content of SMS in asphalt. Compared with the aged asphalt, the content of LMS and SMS in the B3 sample decreased, whereas the content of MMS increased. In addition, it can be seen that the B3 asphalt medium- and SMS had a significant reduction compared with the matrix asphalt. This property helps to reduce the volatilization of small and medium molecular weight substances in the heating and mixing process of recycled, modified asphalt. Therefore, the B3 asphalt has better aging resistance and less asphalt smoke in the use process. In the table, according to reference [41], the molecular weight dispersion of asphalt can be characterized by the ratio of the weight average molecular weight Mw to the number average molecular weight Mn (Mw/Mn). It can be seen from the table that the molecular weight dispersion of aged asphalt increases, whereas the molecular weight dispersion of recycled, modified asphalt is higher than that of base asphalt and lower than that of aged asphalt. This indicates that the molecular weight dispersion of sample B3 is improved compared with aged asphalt.

**Table 12:** Molecular distribution and polydispersity of different asphalts.

Types of asphalt	Proportion of molecules (%)			Dispersion Mw/Mn
	LMS	MMS	SMS	
Virgin asphalt	16.1	56.2	27.7	2.41
Aged asphalt	22.0	56.1	21.9	2.81
B3	20.5	58.4	21.1	2.62

### Correlation between chemical properties and rheological properties

**Fig 19:**
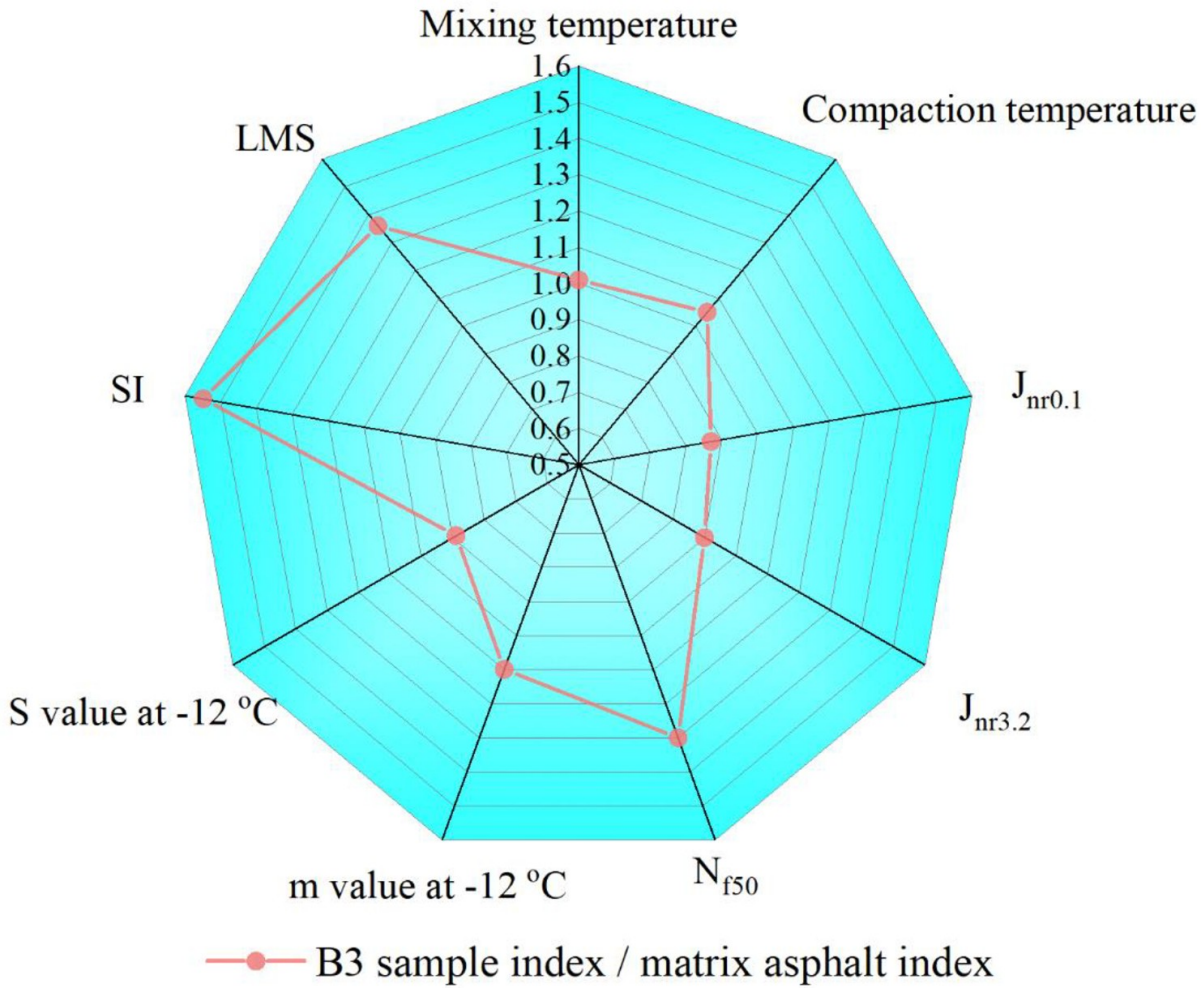
Rheological and chemical indicator comparison of virgin asphalt and B3 asphalt.

Aging increases the content of LMS in asphalt and produces a strong polar group S = O, which enhances the interaction between asphalt molecules and leads to embrittlement. As shown in Fig 19, the rheological and chemical indices of the B3 sample and base asphalt are compared and analyzed. It can be seen from these results that when the mixing and compaction temperatures are close, the J_nr0.1_ and J_nr3.2_ of B3 are lower than those of the base asphalt and the N_f50_ index is higher than that of the base asphalt. This indicates that B3 has better high-temperature performance and fatigue resistance, which is related to the presence of more sulfoxide groups and macromolecules. Sulfoxide groups and macromolecules enhance the intermolecular force of the B3 asphalt, thus improving its performance. The rutting resistance of asphalt at high temperatures also enhances its deformation recovery ability under repeated loading at medium temperatures. Also, although the SI and LMS of recycled asphalt cannot be restored to the level of base asphalt, vegetable oil waste and recycled PE regenerated asphalt shows better road performance.

## Conclusions

1. Based on the rheological index of base asphalt, the optimal additive content of vegetable oil waste and recycled PE regenerated asphalt was determined as 15% R-oil and 4% LDPE.
2. When N_f50_ was used as the fatigue performance index of the asphalt binder, the fatigue life of the vegetable oil waste and recycled PE rejuvenated asphalt was longer than that of base asphalt, which was due to the higher elastic component and stronger deformation recovery ability of the rejuvenated asphalt.
3. FTIR and GPC tests showed that aging increased the content of carbonyl, sulfoxide, and macromolecules in the asphalt, and that R-oil could dilute the content of sulfoxide and macromolecules in aging.
4. A comprehensive comparison of properties between virgin asphalt and the B3 sample showed that R-oil could effectively restore the performance of aged asphalt to the level of base asphalt, whereas the addition of LDPE improved the road performance of recycled asphalt, making the pavement performance of vegetable oil waste and recycled PE slightly better than that of matrix asphalt. Therefore, applying vegetable oil waste and recycled PE to the regeneration of aged asphalt is feasible.
